# Reading out bodily cues to predict interactions

**DOI:** 10.1038/s41598-025-01224-7

**Published:** 2025-05-29

**Authors:** Edoardo Arcuri, Martina Ardizzi, Vittorio Gallese

**Affiliations:** 1 Department of Medicine and Surgery, Unit of Neuroscience, University of Parma, Via Volturno, 39/E, Parma, 43121 Italy; 2 Italian Academy for Advanced Studies in America at Columbia University, New York, USA

**Keywords:** Social intentions, Social affordances, Action prediction, Kinematics, Eye-tracking, Human behaviour, Cognitive neuroscience

## Abstract

Successful motor coordination in social interactions requires the rapid interpretation of others’ intentions from their actions. Previous research suggests that individuals use early bodily cues, such as movement kinematics and gaze, to predict others’ behaviour. However, the motor features critical for signaling or decoding potential motor interactions remain unclear. In this study, we measured the kinematics of a basic motor act — grasping an object — executed with either individualistic (to place) or social (to pass) intentions. Subsequently, we conducted two action prediction tasks to identify bodily markers of (social) intentions. Hand positioning on the object emerged as a key kinematic indicator of the intention to interact with a partner, as shown by kinematic analyses and classification of participants’ responses. Eye-tracking analysis revealed the face as the most attended feature during action observation. Notably, these cues were more consistently attended to when observing actions from a frontal — second-person — perspective rather than a lateral — third-person — perspective. Our findings highlight the saliency of hand-object interactions and the face in decoding potential engagement in second-person contexts. They also provide novel evidence for social affordance processing, expressed in action execution and observation, related to potential motor interactions with others. These features in decoding potential engagement in motor interactions.

**Supplementary Information:**

The online version contains supplementary material available at 10.1038/s41598-025-01224-7.

## Introduction

Interpersonal motor interactions constitute a substantial part of our daily engagements with others and represent a primary form of social interaction widespread among animals^1–3^. In these contexts, others’ bodily displays act as salient stimuli which serve as powerful social cues providing information about others’ intentions^4,5^ and internal states^6,7^ affording for adaptive interpersonal coordination. Proper decoding of these cues can drive crucial social phenomena like sensorimotor synchronization – which is important for learning^8^ and social affiliation^9^ – and can be relevant for monitoring others’ behaviour in both cooperative and conflictual contexts^10,11^. Notably, interpreting others’ behaviour and preparing appropriate motor responses occur on a timescale ranging from hundreds of milliseconds to a few seconds. How, then, is such efficient action prediction during interaction achieved in daily life?

A major line of research in the last three decades has focused on understanding cognitive processes related to observing actions performed by others^12^. Watching other people’s bodily displays is known to activate a broad network of brain sensorimotor areas, including motor areas related to action control^13^, and to impact several physiological and behavioural responses related to motor preparation (see motor resonance phenomena^14^), suggesting a tight functional link between the perception and expression of bodily actions. This fostered early speculation that individuals’ implicit knowledge related to the contextual control of biological motion could be redeployed to aid the interpretation and prediction of others’ actions^15,16^.

Humans’ grasping behaviour represents a paradigmatic case in this regard. The upper limbs’ biomechanic constraints efficient motion to regular kinematic patterns, such as the characteristic bell-shaped velocity profile and end-effector motion trajectory displayed by goal-oriented grasping movements^5^. Fine control of digits is another crucial feature of humans’ manipulative skills, as reflected in preparatory phenomena of grip configuration to the shape, texture, and size of the object of interest^17^. Importantly, these kinematic regularities are substantially influenced by an agent’s intention and can be used to adapt the speed, trajectory, timing, and shape of reaching and grasping actions to achieve specific goals^17–23^.

Some evidence^11,24–27^ suggests that these kinematic modulations can be detected and used to infer motor intentions and predict action outcomes (but see^28^). Furthermore, studies demonstrated that viewing the same arm movement executed with different intents modulates reaction times associated with motor preparation^29,30^ and autonomic responses^31^, indicating early sensorimotor processing of others’ movements. Neural activity patterns in parietal and frontal premotor areas, which host mirror neurons involved in the execution and observation of actions^32,33^, show significant modulation in response to the same motor action executed with different final intentions^34–38^, highlighting the role of perceived kinematic features in the recruitment of motor resources linked to predictive processes^39–41^.

Crucially, the social context and the possibility of interaction significantly influence observers’ behaviour. For instance, interactive settings modulate the visuomotor interference effect, where observing congruent or incongruent actions facilitates or hinders one’s action preparation, respectively^42,43^. Notably, this effect diminishes or even reverses when individuals prepare complementary actions in interactive contexts^44,45^. Similarly, ‘Second-person Neuroscience’ approaches in ecological settings revealed that direct or potential interpersonal engagement alters neural resource involvement^46,47^ and behavioural patterns associated with decoding others’ intentions^48,49^, providing evidence that the detection of social intentions in observed bodily displays has a strong impact on the processing of others’ actions.

Action prediction and preparation during interpersonal motor interaction rely on multiple bodily cues. For example, the gaze direction can reveal crucial information about an action goal by providing indications of where and to what attention is being directed^50^, together with signalling potential interactions^51,52^. Observing someone simply staring at an object evokes behavioural and neural responses similar to those triggered by performing a grasping action toward it^53^, suggesting that gaze can elicit representations of upcoming actions^54^. Additionally, others’ gaze direction modulates neural activity in premotor areas^55^ and visual neurons selective to biological motion^56^, highlighting the close neural link between action and gaze.

Studies using spatial occlusion have explored the impact of different bodily effectors on action prediction. Sartori et al.^24^ found that while participants could discriminate subtle kinematic differences in arm movements, occluding the face in videos of reach-to-grasp actions impaired fine social intention decoding. In another study, Vaziri-Pashkam et al.^57^ showed that both the face and arms provide preparatory cues, enabling faster reactions to a partner’s movements compared to control stimuli like moving dots.

Altogether, these data suggest that early bodily cues such as those expressed by action kinematics or gaze movements significantly influence the beholder’s behaviour. However, the features of others’ actions and social cues critical for signalling or decoding potential interactions remain not fully understood. Moreover, the impact on predictive abilities related to potential direct involvement in interaction has yet to be systematically investigated.

**Fig. 1:**
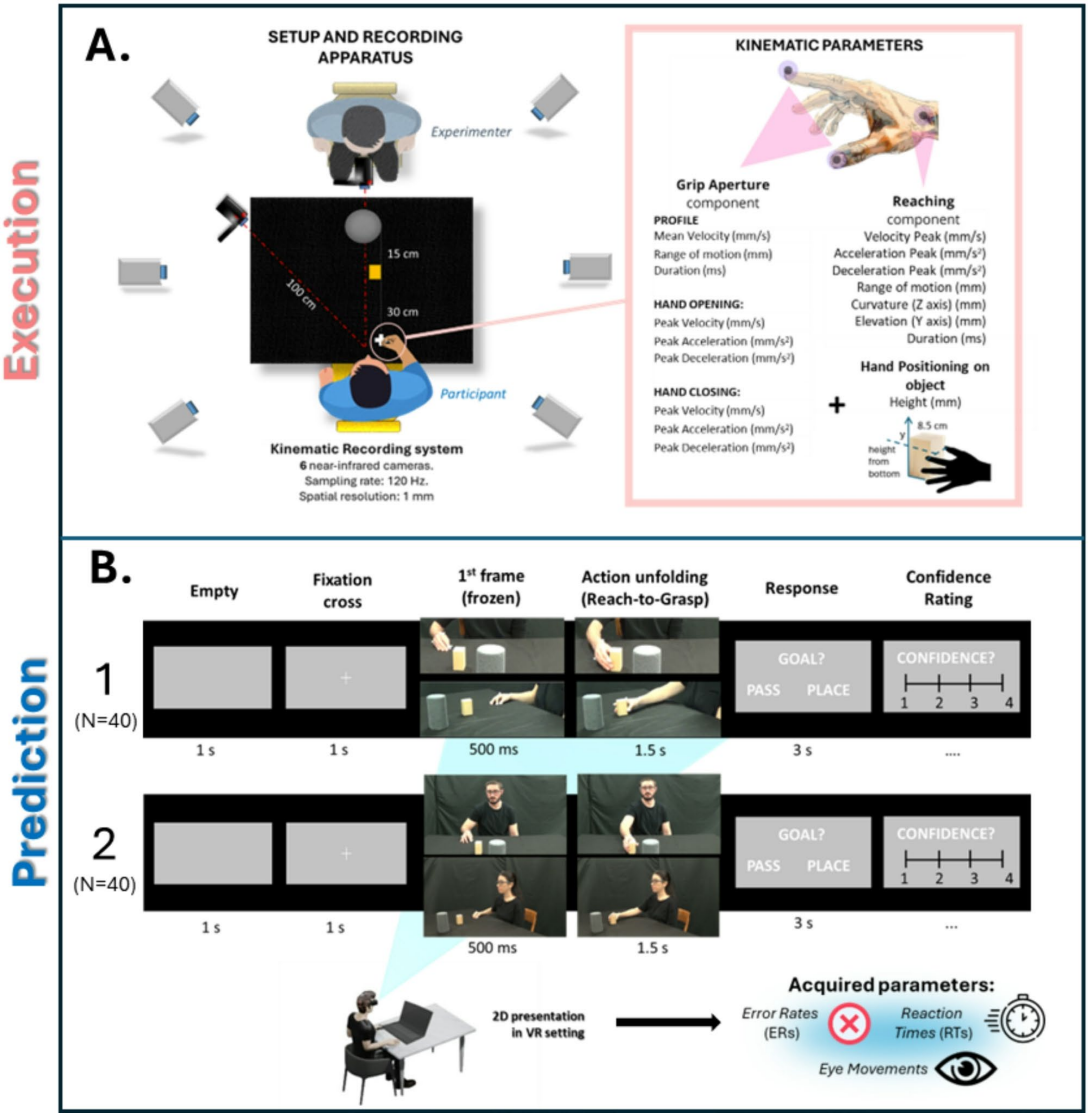
Experimental setting. **(A)**  *On the left*: experimental setup and recording apparatus for kinematics. Participants sat at a table with their right hand in a ‘pinch grip’ posture. The left arm was resting straight at their side. Participants were asked to lean against the table with their chests to prevent wide torse movements, keeping their backs straight and leaning against the chair. The fingertips of the right thumb and index were positioned on a tape-marked point on the table signaling the starting position, located 15 cm to the right of the participant’s mid-sagittal plane and 10 cm from their chest. A small wooden block (5.3 × 4.3 × 8.5 cm) was placed on the Table 40 cm from the chest and 15 cm from a cylinder (9 cm in diameter) serving as the placement base on the table. *On the right*: kinematic parameters of interest for the analysis. Grasping was measured as thumb-finger distance dynamics during hand opening and closing while reaching was obtained by measuring the dislocation of a marker applied to participants’ wrists. Kinematic parameters of interests. **(B)** Single-trial structure and experimental setting for the Action Prediction tasks.

To address this, we adopted a comprehensive approach and conducted two studies (Action Prediction 1 and 2) with different participant groups. Building on previous literature, we focused on the observation of a simple motor act, i.e., grasping an object, performed with either an individualistic goal (grasp to place) or a social goal (grasp to pass). Stimuli were drawn from an independent cohort whose hand and arm kinematics were recorded during an action execution task and later analyzed (Fig. 1A). In line with the previous literature, we expected reach-to-grasp kinematics to differ significantly depending on the social nature of the action^20,58,59^.

In the two Action Prediction studies, we employed a time-constrained dual-choice task, in which participants viewed videos of the reach-to-grasp phase and had to quickly predict the action’s outcome and rate their confidence (see Fig. 1). Signal Detection Theory (SDT) parameters were used to evaluate their ability to infer social intentions from kinematics. Reaction times (RTs) and accuracy were analyzed to assess differences in responses to actions presented from either a 2nd-person or 3rd-person perspective, that is directed toward or away from the observer (Fig. 1B).

In the first study (Action Prediction 1), we aimed to probe the ability to predict actions from the sole observation of the arm movement. To precisely assess how intention and action are perceived based on the kinematic characteristics of the action, we replicated the approach used by Cavallo and colleagues^25^. We employed classification tree analyses to investigate whether there is a relevant subset of kinematic features whose thresholds correlate with perceiving an action as either socially directed or not.

In the second study (Action Prediction 2), participants viewed the same actions but with the entire scene visible, including the agents’ faces. Eye-tracking was employed to determine which effector (e.g., face or arm) was most attended to and how this related to performance. Based on prior findings^24,57^, we expected the face to be highly attended and enhance participants’ decoding performance.

## Results

### Kinematic correlates of social motor intentions

We compared placing actions executed while being alone to passing actions directed toward a partner. For the latter condition, however, we asked participants to randomly alternate between passing or placing actions (see"Methods","Design and Experimental Procedure"). This allowed us to measure the effect of the social context alone – being in the presence of another person – in our experimental setting, while isolating features specific to the intention to interact.

Our Linear Mixed Model (LMM) analysis on seventeen parameters of interest revealed that reaching and grasping in the social context, regardless of the action goal, resulted in overall slower execution and shorter spatial patterns. In thesocial condition, participants were monitored by a partner during both passing and placing actions, likely inducing heightened motor control and accuracy^60^. However, after applying correction for multiple comparisons, this difference remained statistically significant only between placing while being alone and passing. Overall, grip and reaching dynamics had longer durations when the partner was present (blue rows in Table 1), while all others significantly modulated parameters – which regarded range of motion, peak and mean velocity, acceleration and deceleration – were higher for placing actions in the non-social context (red rows in Table 1). Hand position on the object at the time of contact (last row in Table 1) was significantly lower when passing compared to placing while alone. A similar trend, though not significant after correction, was observed for placing in the social context. This suggests that, unlike other parameters which followed a modulation in the direction of the passing action, hand position appeared to be specifically influenced by the agent’s intention to interact rather than the social context alone.

**Table 1:**
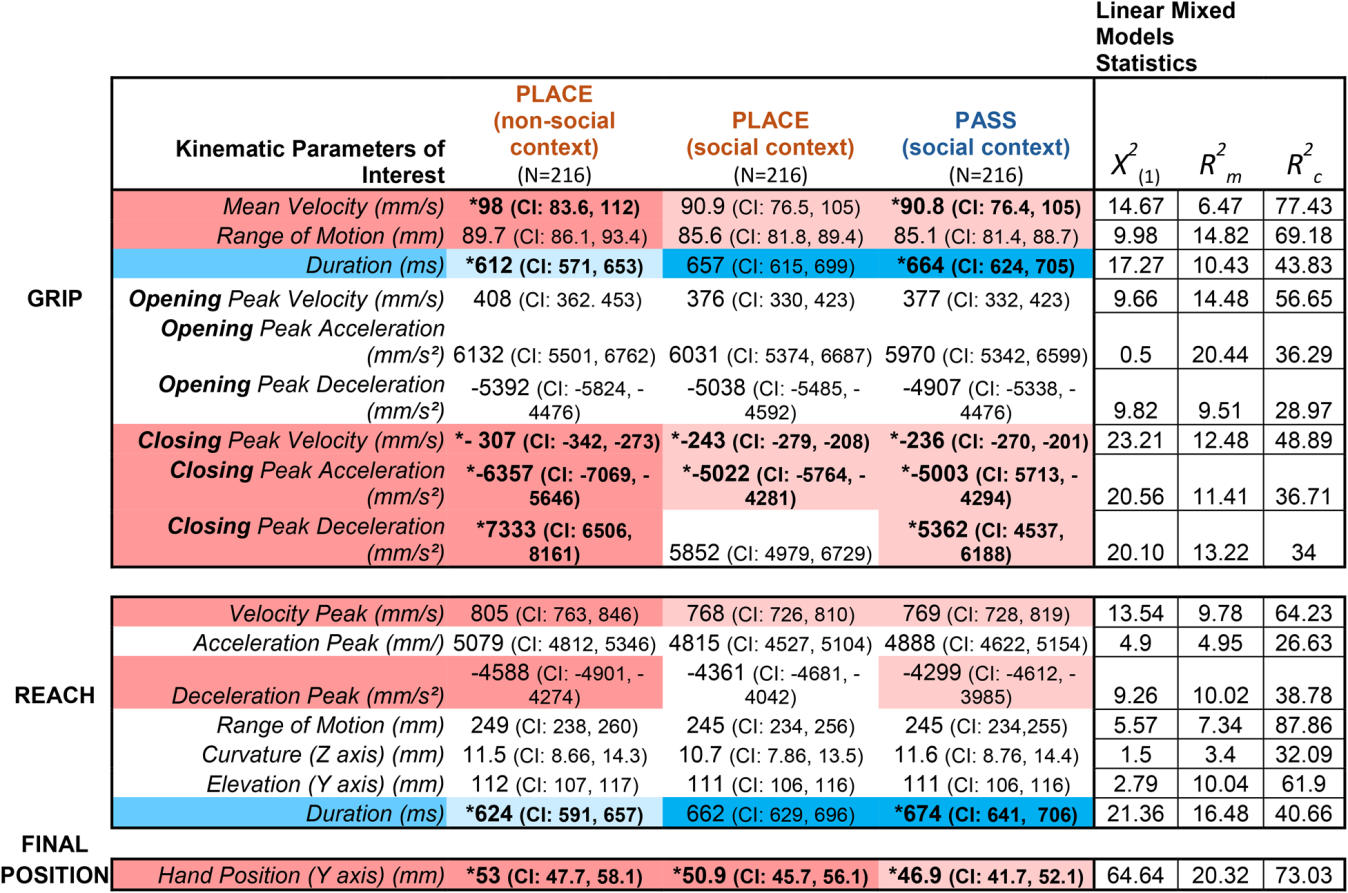
Main results of the kinematic analysis. Table reporting main results from the LMM analysis on kinematic parameters of interest. Means and confidence intervals (CI) for each condition of the execution task are reported for each parameter. Chi-square value is reported along with marginal ($R^{2}_{m}$) and conditional ($R^{2}_{c}$) coefficients of determination used as measures of effect size. Statistically significant results from the individual LMMs are color-coded: parameters highlighted in Pink are significantly higher for at least one placing condition, while those highlighted in light blue are significantly higher for the passing condition. Darker and lighter coding highlight differences among all three conditions, indicating the trending of the modulation. Results which passed bonferroni correction (α/17 = 0.002) are starred (*) and bold, while non-significant results are white. P.

As stimuli for the subsequent Action Prediction experiments, we selected a subsample of placing actions executed alone and passing actions to capture the full range of kinematic differences between these two goals. This choice was guided by the observation that the socially induced slowdown we identified aligns with previous studies on kinematic adjustments in cooperative actions, such as object passing or joint actions^10,58,59,61^, as well as studies suggesting that this regulation may begin in very early developmental stages^62^. By doing so, we aimed to evaluate the extent to which different kinematic components — grasping, reaching, and hand-object interaction — are associated with potential interaction during action observation. To achieve this, we selected a subset of the most representative actions, minimizing within-intention variability (see"Methods","Stimuli Selection"). This process resulted in 96 unique videos depicting the reach-to-grasp phase, evenly distributed between PASS (*n* = 48) and PLACE (*n* = 48) actions.

### Decoding of social actions from kinematics (Action prediction 1)

#### Signal detection analysis

**Fig. 2:**
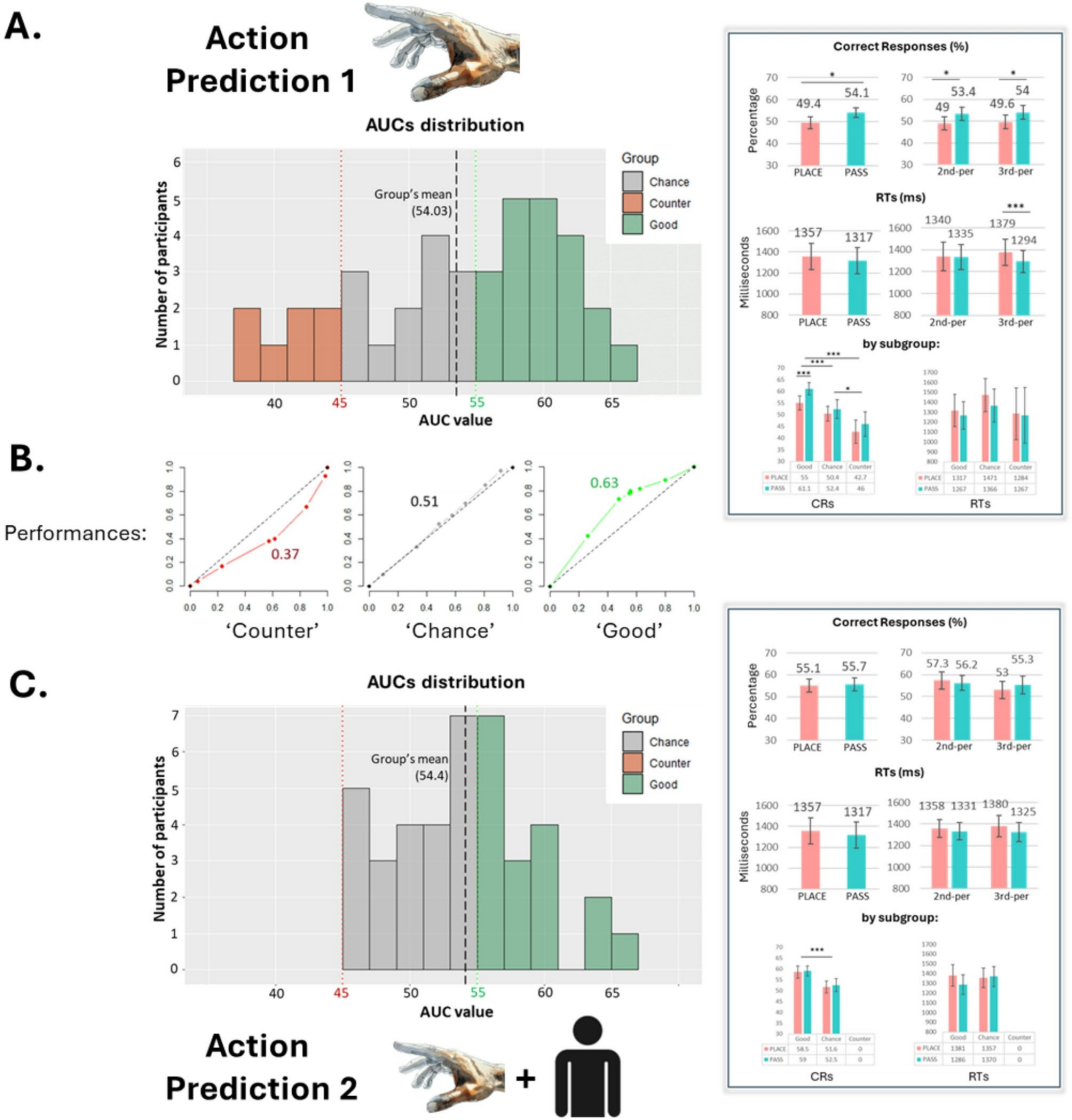
Participants’ decoding performance. **(A)** Results from Action Prediction 1 - arm only; **(C)** Results from Action Prediction 2 – arm + face. *On the left*, histogram representing the distribution of AUCs value among participants. Participants with an Area Under the Curve (AUC) value equal to or above 55 were labelled as ‘good’ decoders, participants with an AUC value between 55 and 45 were categorized as decoding by ‘chance’, and individuals with an AUC value below 45 were designated as ‘counter’ decoders. Categorization by performance is highlighted by color coding. Green = ‘Good’ subgroup (> 55); Grey = ‘Chance’ subgroup (45–55); Red = ‘Counter’ subgroup (< 45). *On the right*, panels reporting bar plots of participants’ % of correct responses and RTs for Goal and Goal + Perspective factors, at both the group (first and second rows) and subgroup (third row) level. Plots in panel **(B)** show individual AUCs of exemplar participants from each of the three subgroups, following the histogram colour coding.

Participants’ ability to discriminate social intentions from early kinematics was evaluated using the Area Under the Curve (AUC) scores derived from SDT (See Fig. 2A). Although AUC values were consistently above chance [$t_{(39)}$ = 2.976, *p* = 0.004, CIs: 51.59–56.47], mean group performance was modest (54.03). Of the 40 participants, only 20 resulted in ‘good’ decoders – performing fairly above the chance level (> 55). 13 participants performed around chance level, while 7 participants showed a counter-decoding pattern. This means that, rather than performing randomly, they systematically misclassified one action intention for the other (e.g., PASS as PLACE, as PASS actions were considered true values for computing the ROC). Thus, while only half were effective intention decoders, most participants (27/40) demonstrated sensitivity to kinematic differences.

#### Linear mixed model analysis

LMM analysis of correct responses [$R^{2}_{m}$ = 20.37, $R^{2}_{c}$ *=* 54.07] and RTs [$R^{2}_{m}$  = 13.29, $R^{2}_{c}$ *=* 41.47] revealed that PASS actions were identified more accurately [t_(9526)_ = 2.20, *p* = 0.04] than PLACE actions (Fig. 2A), with a trend for faster responses for PASS actions that lost significance after post-hoc correction [$X^{2}_{(1)}$  = 9.34, *p* = 0.002; $t_{(9256)}$ = 1.6, *p* = 0.09]. Responses to PASS actions were also faster in the 3rd-person perspective condition [$X^2_{(1)}$ = 8.32, *p* = 0.003; $t_{(9256)}$ = 2.78, *p* = 0.005], though accuracy did not improve. In line with our classification, subgroup performance influenced accuracy [$X^2_{(2)}$ = 34.76, *p* < 0.000], with the ‘good’ group performing best [$t_{(9256)}$ = 6.72 vs. ‘chance’, 12.92 vs. ‘bad’, all Ps < 0.001].

Predictably, a main effect for the Confidence Rating factor was found for RTs [$X^2_{(3)}$ = 143.39, *p* < 0.000], showing that the quicker the response, the higher was the confidence rate indicated by participants (see Supplementary Material). However, confidence level did not significantly affect participants’ accuracy [$X^{2}_{(3)}$ = 4.17, *p* = 0.24]. The LMM on confidence ratings [$R^{2}_{m}$ = 5.28, $R^{2}_{c}$ *=* 26.71] showed that confidence did not differ across subgroups, goals, or perspectives (all ps > 0.06). A significant interaction was highlighted between Group and Perspective [$X^{2}_{(2)}$ = 7.15, *p* = 0.02], showing that ‘counter’ decoders expressed more confidence in the 2nd-person perspective [$t_{(9254)}$ = 2.47, *p* = 0.01]. Further analyses on confidence are reported as Supplementary Material.

#### Classification analysis

**Fig. 3:**
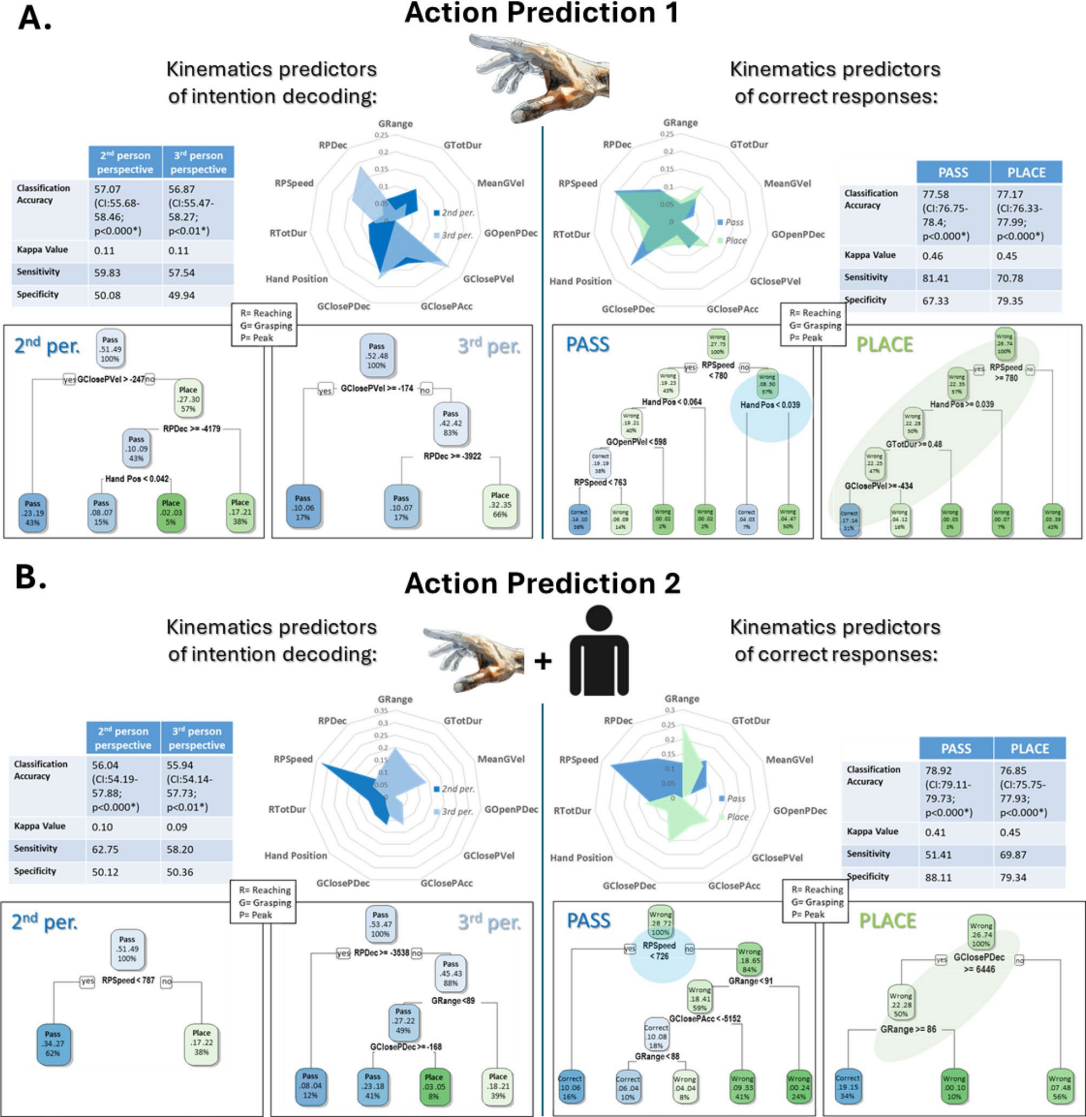
Classification analysis of participants’ responses. **(A)** Results from Action Prediction 1 - arm only; **(B)** Results from Action Prediction 2 – arm + face. Polar plots represent the relative weight of each kinematic variable in response prediction, with tables reporting parameters of classification performance. Decision trees beneath show the main classification processes. Labels for parameters of interest: Grange = Grasp Range of Motion; GTotDur = Grasp Total Duration; MeanGVel = Mean Grasp Velocity; GOpenPDec = Grasp Open Peak Deceleration; GClosePVel = Grasp Close Peak Velocity; GClosePAcc = Grasp Close Peak Acceleration; GClosePDec = Grasp Close Peak Deceleration; RTotDur = Reaching Total Duration; RPSpeed = Reaching Speed; RPDec = Reaching Peak Deceleration; Hand Pos = Hand Position.

A series of classification analyses (see "Methods", "Statistical analyses") examined the relationship between participants’ responses and the features of observed actions. We first assessed whether kinematics predicted participants’ responses. (Fig. 3A, right left panel). Classification of participants’ responses from stimuli kinematics was significantly above chance level with fair although not strong accuracy (~ 57%), possibly reflecting the group’s inconsistent performance. Indeed, greater classification scoring and stronger reliability and specificity were obtained for the ‘good’ and ‘counter’ subgroups, confirming greater within-subgroup coherency (see Supplementary Fig. 1 A). As displayed in the left polar plot in Fig. 3A, hand-related parameters were the strongest predictors in the 2nd-person perspective. In particular, a cluster of grasping parameters related to hand-object approach, including closure parameters (“GClose”) and final hand placement, held the major relevance. Notably, low grasping deceleration and low hand placement (associated with slow reaching deceleration) increased the likelihood of classifying an action as social, as highlighted by the decision tree. Instead, in the 3rd-person perspective, closure parameters and reaching deceleration, but not hand positioning, were the key predictors.

Classifications of subgroups revealed that for both the ‘good’ and the ‘counter’ groups, choice was mainly guided by global, speed-related parameters like grasping total duration, velocity, and reaching speed, which our PCA showed as the main overall indicators of kinematic variations. However, the ‘counter’ group decoded intentions oppositely from the ‘good’ group: actions with a wider grip and faster speeds were more likely to be decoded as social (see Supplementary Fig. 2 A, right panel). Interestingly, the only parameter that was not reverted were the reaching deceleration and the hand positioning on the object: when hand position was low (Hand_Pos < 0.037 mm), even the ‘counter’ subgroup attributed a social intention to the action observed, in line with group level.

Finally, the classification of correct responses from kinematics (Fig. 3A, right panel) showed high accuracy (~ 77%), specificity (~ 67%), and sensitivity (~ 81%), demonstrating consistent kinematic profiles for correct responses at the group level (confirmed by subgroup analyses, Supplementary Fig. 1B). Reaching speed, hand position, and grasping velocities were key predictors, with hand position playing a stronger role in decoding social actions. Decision trees revealed that low hand position alone was sufficient for correctly decoding social actions, while this was not the case for placing actions.

### Saliency of bodily effectors (Action prediction 2)

#### Signal detection analysis

Contrary to our hypothesis, the group displayed a similar performance to the one from Action Prediction 1. Again, the distribution of AUC values was slight, although consistently, above chance level [$t_{(39)}$ = 4.94, *p* < 0.000, M = 54.4 CIs: 52.62–56.50]. However, the distribution of AUC values notably differed from the first study, as no participants displayed a ‘counter’ performance (see Fig. 2C). 23 participants performed at the chance level, while the remaining 17 were good decoders.

#### Linear mixed model analysis

LMMs of accuracy [$R^2_{m}$ = 20.21, $R^2_{c}$ *=* 26.56] and RTs [$R^2_{m}$ = 9.36, $R^2_{c}$ *=* 33.98] showed less efficiency than in Action Prediction 1, as reflected by the lower $R^2$ values, possibly due to increased chance-level performances. Differently from Action Prediction 1, no significant effects for RTs emerged for either the action goal or perspective [all ps > 0.17]. However, a significant interaction was found between Group and Goal [$X^2_{(1)}$ = 11.62, *p* < 0.000] for RTs, as ‘good’ decoders responded faster to passing actions than to placing actions [$t_{(59.7)}$ = 94.9, *p* < 0.001] (Fig. 2C). Performance [$X^2_{(1)}$ = 29.31, *p* < 0.000] reflected our classification, with the ‘good’ group significantly outperforming the ‘chance’ group in accuracy [$t_{(59.7)}$ = 3.30, *p* < 0.01].

Confidence had a significant effect on accuracy [$X^2_{(3)}$ = 10.07, *p* = 0.01], with higher ratings (3 and 4) corresponding to higher accuracy [Accuracy for Confidence Ratings: 4, M = 61.7, CIs = 56.1–67.3; 3, M = 56.2, CIs = 52.4–60.1; 2, M = 52.3, CIs = 48.3–56.2; 1, M = 51.4, CIs = 46.3–56.5. All Ps < 0.02 for Post-Hoc contrasts for 3 and 4 vs. 1 and 2]. Ratings [$R^2_{m}$ = 5.07, $R^2_{c}$ *=* 15.80] for the PASS goal were more confident than for the PLACE goal [$X^2_{(1)}$ = 7.82, *p* = 0.005; PASS: M = 2.54, CIs = 2.42–2.65; PLACE: M = 2.47, CIs = 2.42–2.65; $t_{(9662)}$ = 2.87, *p* = 0.004], and the 2nd-person perspective was rated with more confidence than the 3rd-person perspective [$X^2_{(1)}$ = 7.75, *p* < 0.005; 2nd: M = 2.54, CIs = 2.43–2.65; 3rd: M = 2.47, CIs = 2.36–2.58; $t_{(9662)}$ = 2.53, *p* < 0.01]. Trials in the 2nd-person perspective were more accurately detected at the highest confidence level [Confidence × Perspective interaction: $X^2_{(3)}$ = 9.61, *p* = 0.02; $t_{(9662)}$ = 3.01, *p* < 0.002]. No significant differences in accuracy emerged from the interaction of confidence and performance [$X^2_{(3)}$ = 6.36, *p* = 0.09].

#### Classification analysis

Similar to Action Prediction 1, the classification of participants’ responses based on kinematics was significantly above chance, though with only moderate accuracy (~ 56%). Unlike Action Prediction 1, where hand-related parameters were the strongest predictors of participants’ choices in the 2nd-person perspective, reaching speed emerged as the primary indicator of action decoding in this condition, with slower reaching speed associated with social actions. Conversely, in the 3rd-person perspective, grasping range of motion and approach-related parameters — such as reaching deceleration and hand-closing deceleration — were the most relevant, with slower deceleration and smaller grip aperture being decoded as social. When classifying correct responses based on kinematics, slow reaching speed was the strongest predictor of accurate social action decoding, whereas high hand-closing deceleration and a broader grasp range of motion were predictive of non-social actions.

#### Eye-tracking analysis

**Fig. 4:**
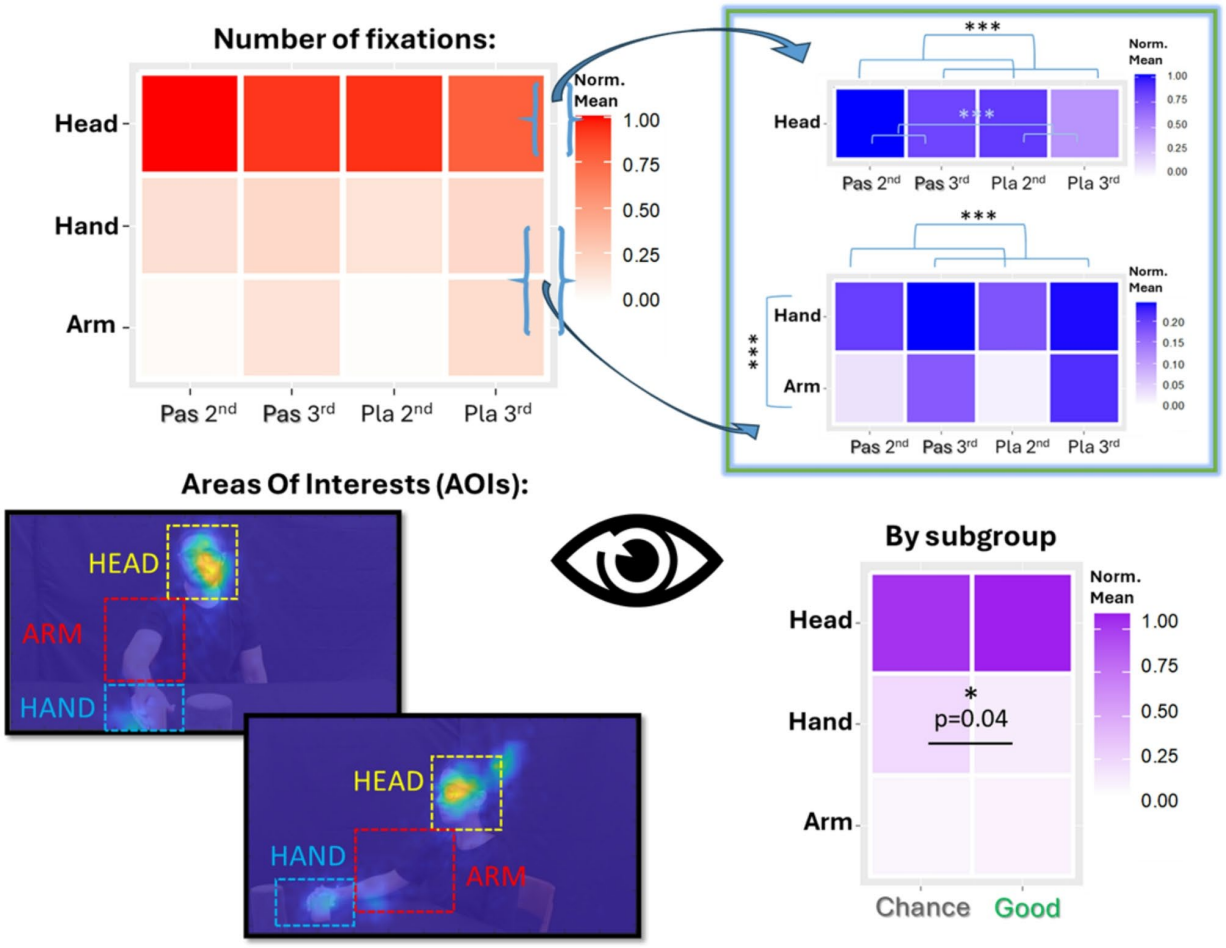
Main results from number of fixation analysis. Results are plotted as heatmaps of normalized mean values of the number of fixations. *Top left*: Heatmap (red) displaying the mean number of fixations for each Area of Interest (AOI) (i.e., Head, Hand, and Arm) across experimental conditions (i.e., Pass 2nd, Pass 3rd, Place 2nd, and Place 3rd). *Top right*: Heatmaps (blue) showing (1) the mean number of fixations on the Head AOI across experimental conditions, and (2) the mean number of fixations on the Hand and Arm AOIs across experimental conditions. *Bottom left*: AOIs for fixation analysis. Rectangular overlays represent the designated AOIs: Face (yellow), Arm (red), and Hand (light blue). AOIs were dynamically adjusted frame by frame. *Bottom right*: Heatmap (purple) showing the mean number of fixations for each AOI across participant groups (i.e., ‘Good’ and ‘Chance’).

Figure 4 shows heatmaps displaying normalised averages of the number of fixations on three Areas of Interest (AOIs): the FACE, HAND, and ARM. LMMs of fixations [$R^2_{m}$ = 31.78, $R^2_c$ *=* 34.98] revealed a main effect of AOI [$X^2_{(2)}$ = 6313.41, *p* < 0.000] and interactions with Goal [$X^2_{(2)}$  = 52.10, *p* < 0.000] and Perspective [$X^2_{(2)}$ = 111.71, *p* < 0.000]. The head received by far the most fixations compared to both the hand [$t_{(24071)}$ = 64.03, *p* < 0.001] and arm [$t_{(24071)}$ = 71.41, *p* < 0.001]. Moreover, it was attended to more in social actions compared to non-social actions [$t_{(24071)}$ = 8.96, *p* < 0.001] and, on average, in the 2nd-person perspective [$t_{(24071)}$  = 6.15, *p* < 0.001]. Both the arm [$t_{(24071)}$  = 7.30, *p* < 0.001] and, to a lesser degree, the hand [$t_{(24071)}$ = 2.90, *p* = 0.05] received more fixations in the 3rd-person perspective. Finally, an interaction between AOI and Group [$X^2_{(2)}$ = 16.20, *p* < 0.000] showed that the ‘chance’ group spent more time fixating on the hand compared to the ‘good’ group [$t_{(47.8)}$ = 2.01, *p* = 0.04] (Fig. 4).

## Discussion

In our study, we aimed to assess the presence and relevance of early markers of socially-oriented behaviour in a simple motor action, i.e., grasping an object. Our first goal was to test if differences in the expression of kinematic parameters between grasping with the intention to interact (i.e., to pass an object to someone else) or not (i.e., to place an object) could be effectively detected by an observer and used to predict the outcome of the reach-to-grasp movement observed. Moreover, we wanted to test: (1) the specific weight of kinematic parameters in driving the perception of a socially-oriented action; and (2) the perceptual saliency of relevant bodily effectors like the face, the arm, and the hand during action prediction tasks.

In our first Action Prediction experiment, we tested whether kinematic modulations of grasping when placing or passing could be used by observers to correctly predict action intentions. Participants could see only the agents’ arms, with no other contextual information provided. Our results indicate that while most participants (27 out of 40) could fairly discriminate kinematic modulations between the two types of actions, intention attribution varied. Specifically, while 20 participants decoded actions consistently above the chance level (> 55 AUC, ‘good’ group), 7 participants, labelled ‘counter’ decoders, had significantly negative (< 45) AUC values, meaning they consistently misattributed action intentions.

In particular, counter decoders tended to attribute higher speed and broader spatial movements to passing actions, which were instead characterised by a slower and shorter motion. Our classification analysis on subgroups ruled out the possibility that such an ‘inversion’ was driven by differences in the relevance of specific sub-components of reaching and grasping in participants’ choice, which could have suggested the adoption of different decoding strategies. Rather, the two groups differed in the decoding of the perceived global shift in speed. Faster rather than slower movements in cooperative actions might happen as a result of social cues that heighten the urgency to respond, like when confronting a partner with a high social status^63^ or when higher attention is paid from another to the agent’s movements^64^. The stimuli we employed, however, were devoid of such contextual information. It is nonetheless possible that differences in personality traits or social behavior influence the perceived sociality of faster movements. For example, higher empathic traits have been correlated with stronger motor facilitatory effects during interactions^65^ and imitation^66,67^, possibly indexing higher reactivity to variations in kinematics, while high autistic traits have been correlated with an altered integration of perceived movement and goals, possibly related to the employment of a more ego-centered motor control scheme^65^. It is known that social anxiety can affect the sensory facilitation of motor responses within one’s peripersonal space^68^, and it is likely that such a trait impacts the perception of others’ actions, especially when approaching the subject. Action observation is also known to be affected by personal motor skills and the similarity in kinematic styles between the observer and the agent^27,69,70^, which might have influenced subjects’ perceptions following their motor styles. However, we did not profile participants in terms of social skills, psychological traits, or motor skills assessment, and previous studies did not adopt systematic control of these variables to provide definitive answers. We therefore suggest that future replication studies or studies employing similar paradigms include these measurements to provide further insights into subjective variation related to intention attribution from action observation.

Despite this variability in our sample, analysis of participants’ accuracy and reaction times showed that socially oriented actions were generally decoded with higher accuracy. Through classification analyses, we assessed which discriminative features determined this result. Parameters related to grip control in approaching the object, such as grip closing speed and deceleration, were among the most relevant predictors of participants’ choices overall. Interestingly, hand positioning on the grasped object was a major predictor of participants’ correct decoding of social actions, as a lower hand placement correlated with perceiving actions as socially directed. Notably, this held for the ‘counter’ group too, which showed opposite intention attribution for other kinematic parameters such as speed and range of motion.

Our kinematic analysis further supports the relevance of hand positioning as a key marker of interaction intent. While most kinematic adjustments — such as variations in speed and spatial patterns of reaching and grasping — were primarily influenced by the social context (i.e., the presence of a partner), hand placement on the object specifically signaled the intention to interact. This finding aligns with research on the well-established ‘end-state comfort effect’ (see^71^) in social interactions, which demonstrates that individuals adjust their hand positioning to facilitate a partner’s ease of use^72^. Crucially, these adjustments are not driven by general social norms, such as politeness, but rather by an expanded action-planning process that integrates the partner’s motor goal (e.g., handling a hammer differently depending on whether it will be used for nailing or simply placed elsewhere)^73–75^. Notably, this behavior emerges early in development, even before the acquisition of explicit ‘theory of mind’ skills^74^.

In our study, participants consistently grasped the object lower when passing it, likely creating space for the partner’s engagement and facilitating smoother interactions. Thus, our findings suggest that when operating together with a partner on an object, the grasping height is a salient feature indicating the space available for potential interaction. Notably, this parameter was more relevant in the 2nd-person than the 3rd-person perspective, despite both conditions providing full visibility of hand placement on the object. The robustness of this prediction among our group hints at a potential social marker related to the expression of movement co-adaptation in motor kinematics^76,77^. This marker may be perceived as a social affordance^78,79^, which research suggests being coded as action classes in parietal-frontal nodes by mirror mechanisms^12,80^. Importantly, this research showed that mirror neurons’ responses during action observation are robustly modulated by the possibility to engage with other agents within the peripersonal space^81^. Our results add to this body of work, demonstrating the robustness of social affordance perception during action observation and its link to action execution in the 2nd-person context.

In our second Action Prediction experiment, a new group of participants completed the same task, but this time, the observed agent’s full body, including the face, was visible. Contrary to our prediction, adding the face did not improve decoding performance at the group level. However, individual analysis showed no counter-decoding, with participants performing either at chance (*n* = 20) or significantly above it (*n* = 19). While the face did not enhance intention decoding, it influenced kinematic discrimination, possibly due to a shift in strategy. Indeed, eye-tracking confirmed the face as the most attended feature, especially in the 2nd-person perspective and for social actions. Classification analysis revealed a shift from fine-grained kinematic cues (e.g., hand positioning and grasp deceleration) to a more global parameter — reaching speed — as the most predictive of participants’ choices and correct decoding of passing actions. Thus, despite fixations on the face, participants likely still gathered peripheral kinematic cues, with overall speed emerging as the most salient when attention was directed to the face. In contrast, in the 3rd-person perspective, participants fixated more on the arm, and reaching-grasping deceleration better predicted choice and correct decoding of placing actions. Given the brief (~ 600 ms) duration of each action, it is likely that less attention could be allocated to fine-grained kinematics. Additionally, splitting attention may have impacted performance, as ‘good’ decoders primarily fixated on the face, while the ‘chance group’ focused relatively more on the hand effector.

Overall, these results suggest the saliency of face-related information for decoding social intentions from action observations, in line with previous studies^24,51,82^. Notably, in our study, there were no differences in head movements or gaze behavior in the video stimuli that could explain the substantial increase in fixations on the head. As discussed in the introduction, previous research using grasping or pointing actions as stimuli has shown that gaze plays a crucial role in predicting action direction and disambiguating intention, suggesting that face cues and hand movements are tightly linked in naturalistic action observation. A previous study^83^ demonstrated that during an emotion discrimination task, where bodily expressions conveyed emotional tone but the head was fully covered, participants fixated significantly on the ‘void’ spot where the face would be, underscoring the strong tendency to integrate facial and bodily cues. Our findings align with this, showing that when the head is visible during action observation, there is a natural reliance on facial cues, likely altering how the entire body is scanned and how kinematic information is integrated.

## Limitations and outlook

Our results call for further investigation and testing to overcome some important limitations of our study. First, participants were tested on a restricted set of stimuli, as done in previous studies employing similar paradigms. While the rationale behind this procedure is to emphasise action differences based on measured averages, this limits the possibility of extending and generalizing results. Some studies have systematically manipulated the available kinematics variability from a set of stimuli, measuring the impact on action prediction performance^25,66^, demonstrating that larger sampling from executed actions significantly impacts prediction accuracy. Moreover, more recent approaches are moving from action categorization based on averaged kinematics to probabilistic mapping of kinematics variation to action goal (and/or cognitive states), probing intention decoding at the single-trial level^84^.

Another limitation of our study is that the Action Prediction tasks employed an explicit measure of action prediction (i.e., guessing the outcome of the action) with a binary forced-choice task not strongly representative of our typical everyday interactions (for an early criticism, see^85^). Studies employing tasks where prediction processes were either measured (and inferred) from physiological parameters or tied to action selection in live contexts demonstrated the relevance of the task in modulating prediction abilities and behavioural responses. Thus, our results need to be further tested in ‘second-person’ settings and with more naturalistic stimuli (see^86^).

Lastly, although we empirically verified the absence of gaze cues and head movements in our action stimuli, we did not employ quantitative measures of whole-body kinematics or any other method to assess subtle postural differences that might have conveyed information about the agent’s intention. This might be relevant, as one study demonstrated that subtle preparatory movements of the head and body correlate with participants’ reactivity to biological motion^57^.

## Conclusions

Interpersonal motor interactions represent a relevant portion of humans’ daily social interactions. Insights on how motor control is implemented at the neural and the behavioural levels suggest that individuals rely on rapid predictive processes of other people’s actions, with research showing that observing the initial phase of a complex goal-oriented action triggers neurophysiological processes related to action prediction and preparation. Moreover, socially oriented actions appear to form a specific class of motor actions which is processed differently from actions executed with individualistic aims.

Our findings highlight social affordance in motor action as a potential marker of social intentions in naturalistic interactions. When preparing to interact, people adapt their movements to make space for the others’ action; in turn, this adaptation is a salient perceptual feature for a partner, potentially allowing for a coordinated motor response. We also highlighted that when observing an action to predict its intention individuals are highly attentive to information from the face of the observed agent, and that the tendency to gather information from the face during action observation is robust even in the absence of ostensive cues. This comprehensive approach sheds light on the articulated interplay between motor actions and social cognition, offering valuable insights into the dynamics of human interaction.

## Methods

The study was approved by the local ethics committee (Comitato Etico dell’Area Vasta Emilia Nord, protocol n. 20577) and was conducted following the principles expressed in the Declaration of Helsinki. All participants from all studies provided written informed consent. Written, informed consent was given by three of the 18 volunteers to publicly use videos and images from the experimental setting for online open-access publication.

### Stimuli production and kinematic analysis (Action execution Task)

#### Participants

18 naïve volunteers (9 males and 9 females, mean age 24.8 ± 0.6 years; range 20–29) participated in the Action Execution task. All participants were right-handed according to the Edinburgh Handedness Inventory^87^. They had normal or corrected-to-normal vision and no previous diagnosis of neurological or psychiatric disorders. The minimum sample size was determined based on prior kinematic studies examining how reach-to-grasp kinematics are modulated by varying motor intentions^11,24,25,27^.

#### Design and experimental procedure

The Action Execution Task comprised two sessions, balanced among participants. In one session, referred to as the PLACE session, participants were seated alone at the table and monitored from behind a curtain through the apparatus for kinematic recording. Participants were instructed to perform a placing action, wherein they reached and grasped the wooden block and then placed it on top of a plastic cylinder positioned in front of them. The PLACE session consisted of 12 consecutive trials. Before each trial, experimenters instructed participants to assume the starting position and begin the task upon hearing a verbal cue such as ‘Go’. After placing, they were instructed to leave the object and get back to the starting position. In the second session, named the PASS session, one of two experimenters (one male and one female, balanced according to participants’ genders) sat across the table from the participants. Participants were tasked with either placing the wooden block on the cylinder (as in the PLACE session) or passing it to the person seated opposite them. To execute the passing action, participants held the block above the cylinder, awaiting the experimenter’s reach. This session comprised 24 trials organized into 6 sets of 4 trials each. To emulate a socially realistic context for the passing action, participants were instructed to autonomously decide, trial by trial, whether to perform a passing or placing action within each set. They were further instructed to perform at least two passing actions and two placing actions within each set, without disclosing their decision to the experimenters. This approach aimed to prevent swift adaptation to the social scenario and the automatic execution of passing actions, as both experimenters and participants had no expectations regarding the forthcoming action. The trial procedure mirrored that of the PLACE session, with verbal instructions for preparation and execution.

Throughout the whole experiment, participants’ performances were filmed with two cameras, one placed in front of them across the table (between the experimenter and the table during the PASS session) and one placed at a 45° angle on their left. Both cameras were placed 1 m away from the participant. Recordings were used for creating video stimuli of passing and placing actions with two visual perspectives, 2nd-person perspective and 3rd-person perspective, later to be used in the Action Prediction experiments.

#### Kinematics recording: apparatus and acquisition parameters

Participants’ hand kinematics were captured using the 3D-optoelectronic SMART system (BTS Bioengineering, Milano, Italy), located at the Unit of Neuroscience of the University of Parma. The system comprises six infrared cameras, which detected three passive reflective markers (spheres of 6 mm in diameter) affixed to the participant’s right hand using double-sided tape: one marker at the radial styloid process of the wrist, another on the thumbnail of the thumb, and the third on the thumbnail of the index finger. Marker positions were sampled at a frequency of 120 Hz with a spatial resolution of 0.3 mm.

The kinematics of the reaching phase were analyzed by extracting the trajectory based on time, focusing on the marker placed on the wrist. For the grasping phase, the analysis involved tracking the distance over time between the markers placed on the thumb and index fingers. Consistent with previous literature^17^ it was assumed that the grasping movement has an initial phase of finger opening, which ends when this opening reaches its peak (maximum finger opening), followed by a closing of the fingers on the object. The onset of the reaching phase was determined as the first of at least three consecutive frames during which the displacement of the marker placed on the wrist along any axis increased by more than 0.3 mm compared to the previous frame. As a criterion to determine the end of the reaching phase, it was requested that 3 consecutive time frames show a wrist displacement lower than 0.3 mm. The calculation was repeated separately for the X, Y, and Z axes, and the frame nearest to the end of the grasping phase. The start of the grasping phase was selected by considering the first of three consecutive frames in which the distance between the two markers placed on the fingers increased by more than 0.3 mm compared to the previous frame. The end of the grasping phase was defined by considering the first of at least three consecutive frames after the start of finger closure in which the distance between the index and thumb was less than 0.3 mm compared to the previous time instant. Analyses were performed with a personal MATLAB code (R2016b). A low-pass Gaussian smoothing filter (sigma value: 0.93) was applied to the recorded data.

In this way, we were able to reconstruct the overall spatiotemporal dynamics of several kinematic curves (see Supplementary Fig. 3) of the reach-to-grasp movement: the Grip Aperture curve (GAc), defined as the distance between the markers placed on the fingers (mm), together with its velocity (VelGAc, mm/s) and acceleration (AccGAc, mm/s^2^) profiles; the Reaching trajectory curves, defined as the movement of the marker placed on the wrist on the x (Rxc), y (Ryc), and z (Rzc) axes, reaching speed (SpeedRc) and acceleration (AccRc) curves defined as the velocity value reached by the marker placed on the wrist and its derivative.

To obtain a precise measure of the influence of different motor intentions on the reaching and grasping phases, 16 kinematic parameters were extracted from the curves and considered for the analysis (see Fig. 1), including 9 parameters for the grip aperture component and 7 parameters for the reaching component. In addition to the spatiotemporal features of the reach-to-grasp movement, the height of the hand position on the object was measured to investigate positioning adjustment (see Fig. 1). Hand positioning was calculated as the mean between the value on the Y axes of the thumbnail and index markers at the time of contact with the object.

#### Statistical analyses

##### Linear mixed model analysis

A linear mixed-effect analysis was performed to detect significant variations between actions. Each kinematic parameter was modelled through a linear-mixed model (LMM). In each model, the fixed independent variables included Condition, Gender (i.e., male and female), and their interaction. The random-effects structure for each model was determined following a Complex Random Intercept (CRI) approach (see^88^) to prevent inflation of degrees of freedom (i.e., pseudoreplication) and reduce the risk of Type I errors. This method balances the benefits of a maximal random-effects model while controlling for overparameterisation, which can cause singularity and non-convergence. Each LMM included participant ID as random intercept (1|ID) plus the intercept of participants interacting with the within-group effect, that is the Condition (1|ID: Condition). Statistical analyses were performed with R (2021), and LMMs were fitted using the *lme4* software package^89^. Results were subjected to a chi-square test with *car* package and corrected post-hoc with Tukey’s using the *emmeans* package^90^. We further applied the Bonferroni method for multiple comparisons^91^ to the results obtained from the kinematic LMMs. We opted for a more conservative correction given the in-principle correlation of the measured variables (e.g., velocity, acceleration, amplitude etc.). Cook’s distance was employed for the analysis and exclusion of outliers and influential cases.

No significant interactions between action goals and gender were detected in our sample [all ps < 0.16]. Only one parameter showed a significant difference between male and female, that is the peak of acceleration in the grasp opening phase which was higher for females [Grasp Opening Peak Acceleration: F: 7058 mm/s^2^ (CI: 6326–7789), M: 4968 (CI: 4239–5698); $X^2_{(1)}$ = 19.16, *p* < 0.000; $R^2_m$ = 20.44, $R^2_{c}$ = 36.29].

### Action prediction tasks

#### Participants

40 naive volunteers (20 males and 20 females, mean age 22.7 ± 3.4 years; range 18–30) participated in Action Prediction 1 while another cohort of 40 naive volunteers (20 males and 20 females, mean age 22.5 ± 3.13 years; range 19–30) participated in Action Prediction 2, for a total of 80 participants. All participants were right-handed according to the Edinburgh Handedness Inventory, had normal or corrected-to-normal vision, and had no previous diagnosis of neurological or psychiatric disorders.

#### Stimuli selection

Representative actions from the action execution task were selected based on the proximity of each action’s kinematic parameters to the average kinematic values for passing and placing actions, respectively. To study kinematic variations in our experimental stimuli, a principal component analysis (PCA) was performed on the kinematic curves for the grasping and reaching components, followed by a Chi-squared test on the resulting components’ values (i.e., coordinates) to assess significant differences between PASS and PLACE actions. Analyses of kinematic curves were carried out using the built-in R function ‘prcomp’.

The PCA identified grip aperture and reaching speed patterns as the main indicators of overall kinematic variations. These two kinematic curves were most strongly correlated with the first (PC1) and second (PC2) components, which accounted for 54.4% and 22.6% of the total variance, respectively, summing to 76% of the total variance (see Table 2). Additionally, Chi-squared tests followed by Tukey post-hoc analyses showed that the first two components, but not the third, significantly discriminated between the two actions [PC1: X²(1) = 13.9, *p* < 0.000; PC2: X²(1) = 15.3, *p* < 0.000; PC3: X²(1) = 1.9, *p* > 0.5].

**Table 2:** PCA on kinematic curves. For each component, the curve with the highest weight is highlighted in italic.

Curve	PC1	PC2	PC3
GA	*0.3499*	−0.1927	0.0770
VelGA	−0.2597	−0.3179	−0.0806
AccGA	−0.1201	0.2661	−*0.9297*
Rx	−0.1737	−0.0619	0.0494
Ry	0.2322	−0.3924	−0.2176
Rz	0.2299	0.1314	−0.0269
SpeedR	0.0184	−*0.5598*	−0.1425
AccR	−0.1285	0.0035	−0.0415
% of variance	0.5445	0.2262	0.06783
Cumulative %	0.5445	0.7707	0.83855

We then conducted a further quantitative evaluation to select actions, proceeding as follows: (1) PCA results were used to reduce the number of parameters of interest (*N* = 17) for video selection, focusing on parameters most strongly correlated with PC1 (i.e., Grasp Range of Motion, GRoM) and PC2 (i.e., Reaching Speed, RPSpeed) as the best indicators of overall kinematic variations; (2) the proximity of all recorded actions to the GRoM and RPSpeed averages for PASS and PLACE actions was assessed by determining their fit within + 1 and − 0.5 standard deviations from the mean for placing actions and within + 0.5 and − 1 standard deviations for passing actions. This approach slightly emphasized the selection toward the relative direction of the intentional effect (i.e., higher speed and longer spatial patterns for placing, and the opposite for passing) while maintaining potential overlap (see Supplementary Fig. 4). This process yielded 3 passing and 3 placing actions in both 2nd- and 3rd-person perspectives for each of 8 agents, resulting in a total of 96 unique videos.

#### Design and experimental procedure

The 96 selected actions were presented to the participants in video format through the use of a HTC Vive Pro Eye head-mounted display (HMD) (see Fig. 3A). Participants were seated behind a table and were assisted in wearing the HMD. They were asked to keep both hands resting on the table, with the index finger of each hand standing on one of two keyboard buttons to provide a binary response. The experiment was run by employing code in Unity Software (2021.3) for both video presentation in a virtual environment and response collection. In the virtual environment, the participant was positioned in front of a screen in a pitch-black chamber. The screen size and its placement from the participant were recreated to simulate a 27-inch flat screen at ~ 50 cm distance from the participant’s point of view. Responses, Reaction Times (RTs), and eye movement were collected for both experiments.

The design and procedure were identical for both the Action Prediction tasks, differing only in how the 96 actions were presented. In Action Prediction 1, videos were edited to display participants executing the actions solely from below the shoulder (See Supplementary Video S1-S4). In Action Prediction 2, the videos depicted the entire scene, including the participant’s entire upper body and face while acting (See Supplementary Video S5-S8).

At the beginning of the experiment, participants underwent a quick training phase. First, 8 random sample movements were shown to them in sequence, so they could see the phase where agents in the video passed the block to the experimenter or placed it on the cylinder. Subsequently, participants were trained to execute the task and presented with just the reach-to-grasp phase of 8 new videos interrupted at the moment of hand contact with the object to be grasped. For each video, participants were asked to guess the type of motor intention (i.e., passing or placing) underlying the observed action. Only in this phase, trials were followed by feedback, in which the entire video was reproduced.

In both the training and experimental session, each trial (see Fig. 1B) was organized as follows: first, a grey screen appeared for 1 s, followed by another screen displaying a white fixation cross (+) at the centre, also for 1 s. At 2 s, the first frame of one of the 96 videos (presented in random order) appeared, frozen for 0.5 s. Following this pause, the video began playing, concluding within 1.5 s. The end of each action was synched at the time of contact with the object, while the starting of each action could vary according to its velocity. After the video ended, a panel prompted participants to indicate the possible outcome of the observed action (“pass” or “place”, using either the right or the left key, balanced among participants). Participants had a maximum of 3 s to respond; if they did not respond within this time frame, the trial was cancelled, and the next trial commenced. Participants were instructed to respond accurately and promptly. Unlike during the training phase, no feedback was provided after participants’ responses. After selecting a response, participants were required to rate their confidence level regarding their decision on a 4-point scale ranging from 1 = less confident to 4 = more confident (see Fig. 1B) by using the computer keyboard, with no time limit for responding.

The experimental session comprised 3 blocks. Within each block, all videos (*N* = 96) were presented in randomized order, covering both intentions (*N* = 48 for passing; *N* = 48 for placing). In total, 288 trials were administered. The whole experiment had a duration of approximately 40 min.

#### Statistical analyses

##### Signal detection analysis

For each participant, we computed the proportion of hits and false alarms, using PASS actions as the signal and PLACE actions as the noise. Estimated SDT parameters were then integrated with confidence ratings to establish points on an empirical receiver operating characteristic (ROC) curve. Given that each response involved four associated ratings, there were eight potential responses per trial, resulting in seven points on the ROC curve^92^. Subsequently, these points were plotted to determine the area under the curve (AUC) for each participant. Participants’ performances were categorized based on their AUC values (see caption for Fig. 2A).

##### Linear mixed model analysis

To detect differences in the behavioural response to actions executed with an individual (i.e., PLACE) or a social (i.e., PASS) intention from different viewpoints (i.e., 2nd- vs. 3rd-person perspective), LMMs for participants’ RTs and Correct Responses were built with a fully crossed design with fixed factors Goal, Perspective, Performance (i.e., ‘good’, ‘chance’, ‘counter’), and Confidence. Random-effect structure was determined by adopting the CRI approach. Each LMM initially included a full CRI model, including the intercept of participants only plus the intercepts of participants interacting with all nested effects. If convergence or singularity issues arose, which was our case given the complexity of our random-effect structure, we proceeded as in^88^ and iteratively simplified the structure by removing random intercepts. Simplified models that met convergence criteria were then assessed for goodness of fit and compared using Likelihood Ratio Tests and the Akaike Information Criterion (AIC) to select the best-fitting model. Following this procedure, in Action Prediction 1 the selected models for RTs and Correct Responses included random intercepts of the participants and their interactions with Confidence and Goal, respectively. In Action Prediction 2, a more complex random effect structure resulted, which included random intercepts of the participants and of their interactions with each fixed effect (i.e., 1|ID: Goal, 1|ID: Perspective, 1|ID: Performance, 1|ID: Confidence). A third LMM was computed on confidence ratings with fixed factors Goal, Perspective, and Performance. The random-effect structure included random intercepts of the participants and their interactions with each fixed effect.

RTs were calculated on correct responses with a minimum duration of 150 ms as the threshold for physical feasibility^93^. Statistical tests, measurements of effects, and outlier detection were executed in the same way as for the LMM analysis in the Action Execution Task.

##### Classification tree analysis

A series of classification analyses were conducted to explore the relationship between participants’ responses and action features. Specifically:

- The first set of analyses aimed to predict intention decoding from actions’ kinematics. We trained and tested classification trees using significant kinematic parameters identified from the LMM analysis in the Action Execution task as predictors and participants’ choices (i.e., ‘place’ or ‘pass’) as categorical outcomes. Note that we also included parameters that resulted significant before applying the Bonferroni correction, avoiding strong a-priori assumptions about how specific parameters are perceived and used by participants to drive choices. For both Action Prediction 1 and Action Prediction 2, we utilized the entire dataset of trials to observe global patterns. Subsequently, we employed datasets limited to participant subgroups (‘good’, ‘chance’, and ‘counter’) to examine variations in the relevance of kinematic features for action prediction between subgroups.
- The second set of classification analyses aimed to determine which action features were most predictive of participants’ correct responses. For this analysis, correct and incorrect responses were used as categorical outcomes, and the classification was repeated for the entire dataset and each subgroup.

Each classification model was trained with 80% of the selected dataset and tested on the remaining 20%. Classification Trees were constructed using the *rpart* package in R^94^. The complexity of decision trees was determined based on default cost complexity pruning in the ‘rpart’ function, measured as minimization of the Gini’s index, which defined the minimum improvement in the model required at each node, in addition to cross-validation. The relative weight (i.e., importance) of each variable is calculated in the ‘rpart’ function by measuring the improvement added by variables in cost complexity minimization. The significance of classification accuracy for each classification analysis was assessed with binomial testing against chance level performance.

##### Eye-tracking analysis (Action Prediction 2)

To investigate participants’ attention to specific bodily features, we analyzed the frequency and duration of their fixations on three Areas of Interest (AOIs): the Face, the Hand, and the Arm AOIs. The dimensions of the AOIs were calculated based on the actual stimulus dimensions in the UNITY environment and were utilized as regions for fixation localization. AOIs were dynamically adjusted frame by frame by using fixed points of reference for each effector (e.g., head centre, elbow, and fingertips) to be used as centres around which to build the AOIs. Analysis was performed on eye movements recorded during the reach-to-grasp epoch of the video, that is, from the onset of the reaching movement to the end of the grasping movement. Fixation identification was carried out using a detection algorithm based on two-means clustering through a MATLAB R2023a script^95^. To evaluate differences in overt attention to AOIs between conditions, LMMs were built for the number of fixation. AOIs, Goal, Perspective, Performance, and all their interactions were used as fixed effects; participants’ intercepts and their interaction with Perspective and Group were used as random effects.

## Supplementary Information

Below is the link to the electronic supplementary material.

Supplementary material 1 (PDF 786.3 kb)

Supplementary material 2 (mp4 1351.4 kb)

Supplementary material 3 (mp4 1478.0 kb)

Supplementary material 4 (mp4 1428.9 kb)

Supplementary material 5 (mp4 1555.5 kb)

Supplementary material 6 (mp4 2122.7 kb)

Supplementary material 7 (mp4 2024.9 kb)

Supplementary material 8 (mp4 2141.1 kb

Supplementary material 9 (mp4 2066.8 kb)

## Data Availability

The data supporting this study’s findings are available from the corresponding author upon reasonable request.
