# Supplementary material for: Reading out bodily cues to predict interactions
Source: Sci Rep. 2025 May 29;15:18865. doi: 10.1038/s41598-025-01224-7 (PMC12122890; doi:10.1038/s41598-025-01224-7)
Supplement: Supplementary file 1 — Supplementary material 1 (PDF 786.3 kb) [file 41598_2025_1224_MOESM1_ESM.pdf]

Reading out bodily cues to predict interactions

SUPPLEMENTARY MATERIAL

Edoardo Arcuri(*)[1,], Martina Ardizzi[1], Vittorio Gallese[1,2,+]

1. Department of Medicine and Surgery, Unit of Neuroscience, University of Parma, Parma, Italy
2. Italian Academy for Advanced Studies in America at Columbia University, New York, USA
(*) Corresponding author:
Edoardo Arcuri; Via Volturno, 39/E, 43121, Parma (Italy). Telephone: +39-0521-903873; E-mail: edoardo.arcuri@unipr.it

Supplementary material is numbered and reported below according to positions in the main text of the article 'Reading Out Bodily Cues to Predict Interactions'. Legends of supplementary videos can be found at the bottom of this document.

2.2. Decoding of social actions from kinematics (Action Prediction 1)

LINEAR MIXED MODEL ANALYSIS.

Confidence factor results for RTs: [RTs for Confidence ratings: 4, M=1024, CIs=1160-889; 3, M=1241, CIs=1366-1115; 2, M=1440, CIs=1567-1313; 1, M=1645, CIs=1785-1504; all ps<0.000].

Confidence factor interactions in the Accuracy LMMs: Confidence interacted with the Goal factor [$X^2_{(3)}$= 5.22, p=0.02], as trials for PASS actions rated with the maximal confidence rating (i.e., 4 out of 4) were significantly more correct than those for PLACE actions [t(9526)= 8.61, p=0.04]. Confidence Rating also interacted with the Performance factor [$X^2_{(6)}$= 16.18, p=0.01]. The 'good' group responses with the highest level of confidence held the most accuracy [4, M=66.7, CIs=72.4-60.9; 3, M=57.0, CIs=61.3-52.6; 2, M=55.7, CIs=60.5-51.0; 1, M=52.5, CIs=58.8-46.3; for 4 vs. 1,2, and 3, all ps<0.02], while this was not the case for the 'counter' group [4, M=36.2, CIs=44.6-27.9; 3, M=43.9, CIs=50.9-36.9; 2, M=46.9, CIs=54.8-39; 1, M=53.2, CIs=64.8-41.6; all ps>0.09 for every contrast].The 'chance' group did not show significant differences in accuracy throughout levels of confidence [4, M=54.0, CIs=61.2-46.9; 3, M=50.3, CIs=54.5-46; 2, M=51.5, CIs=55.7-47.2; 1, M=49.2, CIs=55.9-42.6; all ps>0.8 for every contrast,]. Notably, the 'Counter' group significantly made more errors than both the 'chance' and the 'good' groups at higher levels of confidence [Confidence rating 3: 'Counter' vs. 'Good', $t_{(9254)}$= 13.10, p=0.005; Confidence rating 4: 'Counter' vs. 'Chance', $t_{(9254)}$= 17.79, p=0.004; 'Counter' vs. 'Good', $t_{(9254)}$= 30.40, p<0.000]. The 'good' group performed significantly better than the chance group at higher levels of confidence [Confidence rating 3: 'Good' vs. 'Chance', $t_{(9254)}$= 6.72, p=0.05; Confidence rating 4: 'Good' vs. 'Chance', $t_{(9254)}$= 12.61, p=0.01]. Lastly, a triple interaction between Confidence Rating, Performance, and Goal factor was found [X2(6)= 77.93, p<0.000]. The 'good' group responses with higher confidence ratings toward PASS actions were significantly more accurate than those for PLACE actions from the same group [PASS: 4, M=78.0, CIs=86-70.1; 3, M=60.6, CIs=66.7-54.4; PLACE: 4, M=52.8, CIs=57.9-47.7; 3, M=55.3, CIs=63.1-47.1; for 4 and 3, all ps<0.01]. The 'chance' group had significantly more accurate answers for PASS actions when they indicated a moderately high level of confidence (i.e., 3 out of 4) [PASS: 3, M=56.9, CIs=63-50.8; PLACE: 3, M=43.6, CIs=49.7-37.2, p=0.002], whereas they had more accurate answers for PLACE actions when they indicated a moderately low level of confidence (i.e., 2 out of 4) [PASS: 2, M=46.5, CIs=52.5-40.5; PLACE: 2, M=56.5, CIs=62.5-52.5; p=0.002]; the opposite was true for the 'bad' group [PASS: 3, M=39.8, CIs=45.7-30.7; PLACE: 3, M=48, CIs=57.8-38.1, p=0.02; PASS: 2, M=46.5, CIs=52.5-40.5; PLACE: 2, M=56.5, CIs=62.5-52.5, p=0.02].

Overall, results from confidence rating are in accordance with effects found for interactions between Performance and Goal in accuracy and RT scores.

CLASSIFICATION ANALYSIS.

Classification Analyses on Subgroups:

A

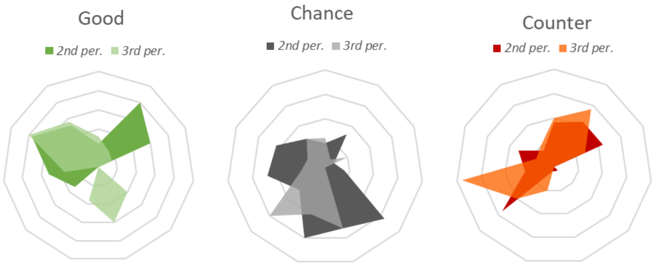

	2nd person perspective	3rd person perspective	2nd person perspective	3rd person perspective	2nd person perspective	3rd person perspective
**Classification Accuracy**	**61.37** (CI:59.19-63.35; p<0.000*)	**63.19** (CI:61.04-65.31; p<0.000*)	52.87 (CI:53.09-55.87; p<0.008*)	53.87 (CI:51.77-55.96; p<0.01*)	**60.16** (CI:56.56-63.68; p<0.000*)	55.47 (CI:51.83-59.06; p<0.04*)
**Kappa Value**	**0.22**	**0.25**	0.05	0.07	**0.20**	0.09
**Sensitivity**	62.99	62.99	54.95	57.01	64.27	62.99
**Specificity**	59.58	59.58	50.81	50.56	56.08	59.58

B

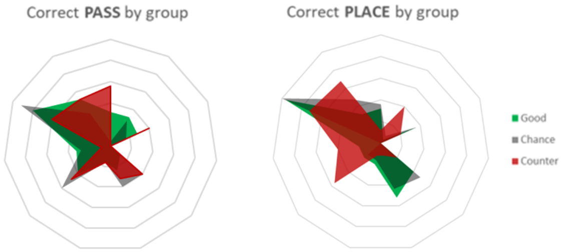

	Good	Chance	Bad
**Classification Accuracy**	**81.29** (CI:80.7-83.1; p<0.000*)	**78.16** (CI:76.91-79.37; p<0.000*)	**80.84** (CI:78.76-82.8; p<0.000*)
**Kappa Value**	**0.60**	**0.46**	**0.33**
**Sensitivity**	80.93	82.11	93.47
**Specificity**	84.12	66.73	34.67

Good	Chance	Bad
**78.04** (CI:76.72-79.31; p<0.000*)	**75.91** (CI:74.62-77.91; p<0.000*)	**79.97** (CI:77.86-81.97; p<0.000*)
**0.47**	**0.33**	**0.32**
81.29	85.88	93.13
69.22	45.94	34.81

Supplementary Figure 1 – The figure shows the main results obtained at the subgroup level in Action Prediction 1. Polar plots represent the relative weight of each kinematic variable (see Fig. 2B in the paper for list and order) in response prediction; tables report parameters of classification performance. (A) reports results for kinematic predictors of subgroups' choices, while (B) reports results for kinematic predictors of correct responses for subgroups.

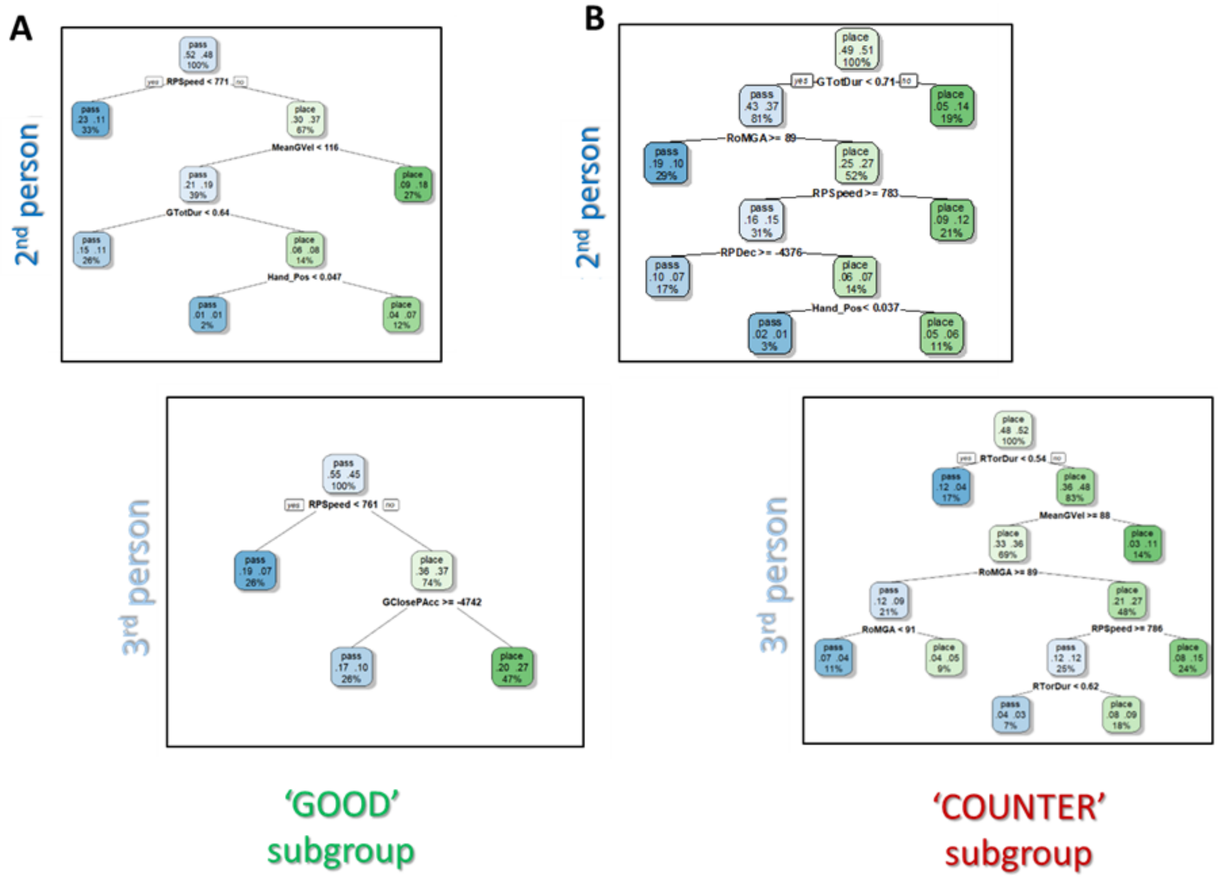

Supplementary Figure 2 – Decision trees for the 'good' (A) and 'counter' (B) subgroups. RoMGA= Grasp Range of Motion; GTotDur= Grasp Total Duration; MeanGVel= Mean Grasp Velocity; GOpenPVel= Grasp Open Peak Velocity; GOpenPDec= Grasp Open Peak Deceleration; GClosePVel= Grasp Close Peak Velocity; GClosePAcc= Grasp Close Peak Acceleration; GClosePDec= Grasp Close Peak Deceleration; RTotDur= Reaching Total Duration; RPSpeed= Reaching Speed; RPDec= Reaching Peak Deceleration; Rdistance= Reaching Distance; Hand_Pos= Hand Position.

6.1.3 Kinematics recording: apparatus and acquisition parameters

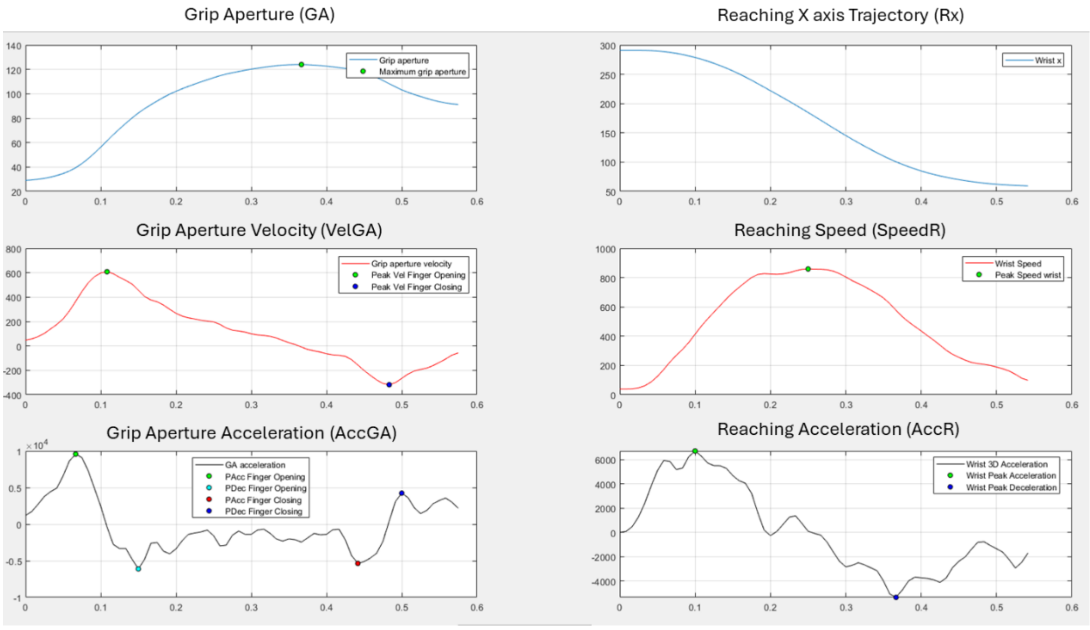

Supplementary Figure 3 – Kinematic Curves. Screenshot of the MATLAB code output showing examples of kinematic curves. The X-axes represent time in seconds, while the Y-axes represent spatial parameters (mm), speed parameters (mm/s), and acceleration parameters (mm/s²).

6.2.2 Stimuli Selection

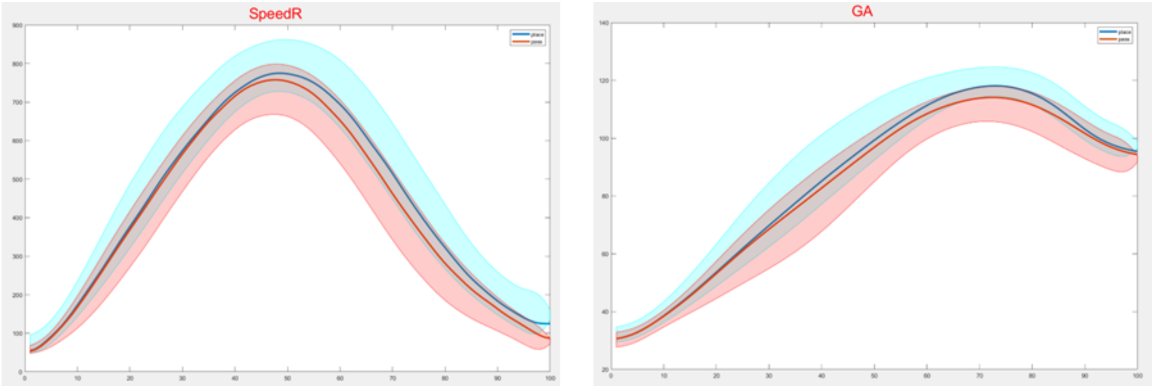

Supplementary Figure 4 – Visualization of the ranges of interest for the selection of representative actions for Pass (light blue) and Place (pink) actions on the Reaching Speed (SpeedR) and Grip Aperture (GA) curves. Thick coloured lines indicate averages. Shaded areas represent standard deviation ranges employed for action selection (+1 to -0.5 for placing actions, +0.5 to -1 for passing actions).

Supplementary Video S1. ACTION PREDICTION 1, PLACE – 2ND PERSON: reach-to-grasp phase of a representative action performed with the intent to place the object on the cylinder, frontal view.

Supplementary Video S2. ACTION PREDICTION 1, PLACE – 3RD PERSON: reach-to-grasp phase of a representative action performed with the intent to place the object on the cylinder, lateral view.

Supplementary Video S3. ACTION PREDICTION 1, PASS – 2ND PERSON: reach-to-grasp phase of a representative action performed with the intent to pass the object to another person, frontal view.

Supplementary Video S4. ACTION PREDICTION 1, PASS – 3RD PERSON: reach-to-grasp phase of a representative action performed with the intent to pass the object to another person, lateral view.

Supplementary Video S5. ACTION PREDICTION 2, PLACE – 2ND PERSON: reach-to-grasp phase of a representative action performed with the intent to place the object on the cylinder, frontal view

Supplementary Video S6. ACTION PREDICTION 2, PLACE – 3RD PERSON: reach-to-grasp phase of a representative action performed with the intent to place the object on the cylinder, lateral view.

Supplementary Video S7. ACTION PREDICTION 2, PASS – 2ND PERSON: reach-to-grasp phase of a representative action performed with the intent to pass the object to another person, frontal view.

Supplementary Video S8. ACTION PREDICTION 2, PASS – 3RD PERSON: reach-to-grasp phase of a representative action performed with the intent to pass the object to another person, lateral view.
